# Toolkits and Libraries for Deep Learning

**DOI:** 10.1007/s10278-017-9965-6

**Published:** 2017-03-17

**Authors:** Bradley J. Erickson, Panagiotis Korfiatis, Zeynettin Akkus, Timothy Kline, Kenneth Philbrick

**Affiliations:** Mayo Clinic, 200 First St SW, Rochester, MN 55905 USA

**Keywords:** Artificial intelligence, Machine learning, Deep learning, Convolutional neural network

## Abstract

Deep learning is an important new area of machine learning which encompasses a wide range of neural network architectures designed to complete various tasks. In the medical imaging domain, example tasks include organ segmentation, lesion detection, and tumor classification. The most popular network architecture for deep learning for images is the convolutional neural network (CNN). Whereas traditional machine learning requires determination and calculation of features from which the algorithm learns, deep learning approaches learn the important features as well as the proper weighting of those features to make predictions for new data. In this paper, we will describe some of the libraries and tools that are available to aid in the construction and efficient execution of deep learning as applied to medical images.

## Introduction

Deep learning is an important new area of machine learning which encompasses a wide range of neural network architectures designed to complete various tasks [1–4]. In the medical imaging domain, example tasks include organ segmentation, lesion detection, and tumor classification [5–8]. The most popular network architecture for deep learning for images is the convolutional neural network (CNN). Whereas traditional machine learning requires determination and calculation of features from which the algorithm learns, deep learning approaches learn the important features as well as the proper weighting of those features to make predictions for new data. In this paper, we will describe some of the libraries and tools that are available to aid in the construction and efficient execution of deep learning as applied to medical images.

### How to Evaluate a Toolkit

There is not a single criterion for determining the best toolkit for deep learning. Each toolkit was designed and built to address the needs perceived by the developer(s) and also reflects their skills and approaches to problems. Therefore in this report, we will attempt to objectively assess each toolkit using a range of different criteria, but in many cases, the assessment is subjective. Therefore, this is mostly a description of the tools. From this, it is hoped that readers can determine the toolkits that are most likely to work well for them.

The criteria we describe include:

#### Language

The computer language that the toolkit was written in can impact the ability to use it effectively. This can be important if you expect you will adjust some parts of the toolkit internals. The language will also impact to at least some degree the language(s) you can use for your development, though many of the toolkits do have bindings or other mechanisms that allow you to access the toolkit from a language different from what it was written in.

#### Documentation

The quality and coverage of documentation for the toolkit as well as examples is critical to enable effective use of a toolkit. Clearly, high quality documentation as well as examples that are similar to problems you will work on will be helpful to efficiently developing a solution to your problem. Good documentation is also a sign that the tool is mature and not changing rapidly.

#### Development Environment

The ease of programming to create a network needs a development environment. This is a highly subjective assessment, and we have tried to identify objective properties that will affect a person’s subjective evaluation. For example, some toolkits have graphical integrated development environment. Some will prefer this while others prefer a dedicated editor and command line. Some have visualization tools for affirming the network is correct; others have visualization tools for monitoring the learning progress.

#### Execution Speed

This is the speed of actually classifying or segmenting the image using a trained network. While it will involve hundreds to thousands of calculations per pixel, this can usually be accomplished in seconds for medical uses. Usually, this is substantially faster than training and is usually much less important than training speed for medical applications.

#### Training Speed

While execution of a trained network can be important in some cases, the training time is usually many orders of magnitude slower. Therefore, training speed is likely to be of greater consequence than execution speed. The training speed depends on how efficient the math libraries are and how well those libraries take advantage of the available computational resources, and also depends heavily on the nature of the task and images. For instance, the memory available to the processor and the bandwidth from storage to processing unit will have a huge effect on performance when training large datasets, especially when all data must be accessed at each iteration. As such, estimation of speed will be a very rough estimate.

#### GPU Support

Graphical processing units or GPUs can significantly increase the rate at which networks learn. Special libraries like cuDNN are an example of how the special type of calculations required for deep learning have been adapted and optimized for computation by a GPU. Most toolkits leverage cuDNN as their way to support GPUs. Some are able to support multiple GPUs with little developer effort, some require more effort, and some simply cannot support more than one GPU. Supporting more than one GPU will often result in substantial performance gains that nearly match the number of cards added. As noted above, the coupling of storage with processing will have a large impact on performance.

#### Maturity Level

This is our subjective estimate of how mature a toolkit is. We estimated this using a combination of a large user base, few bug fixes in the last few months, and a good support community.

#### Model Library

In some cases, toolkits also have a library of code that creates networks, and may even have the weights associated with the nodes. One of the most recognized of these is the Caffe “model zoo”, where one may download many of the popular reference networks and weights.

#### GitHub Commits

This is the number of changes made to the toolkit code since the project was placed on GitHub. This is an objective measure, but can be misleading, as a toolkit may have been developed using some other mechanism, and only recently placed on GitHub. This will result in a low number of commits compared to its true maturity. Conversely, if a toolkit was started in a very early stage or with many bugs, there can be many commits despite a low level of maturity.

#### GitHub Contributors

This reflects how many different people are contributing to the project on GitHub. In general, more contributors reflect a vibrant community with many users and also likely a broader range of users/applications.

## Toolkits

### Caffe

**Fig. 1:**
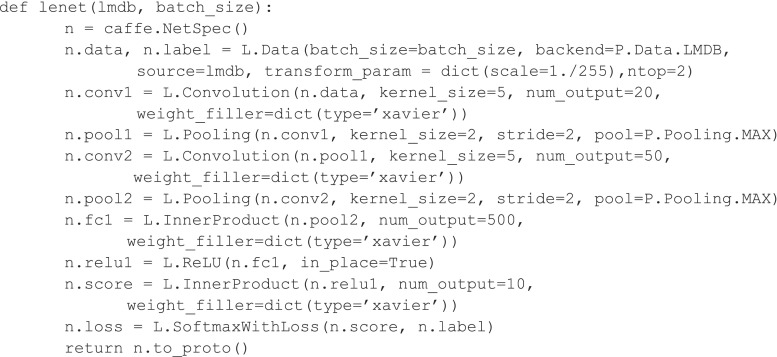
Example code implementing LeNet CNN written in Caffe

Caffe is one of the most mature toolkits, and was developed by Berkeley Vision and Learning Center. It is modular and fast and supports multiple GPUs with little extra effort. It uses a JSON-like text file to describe the network architecture as well as the solver methods. Also has model zoo, which is a website where you can download Caffe models as well as network weights. This can help you get going very quickly with examples. However, tuning hyperparameters is more tedious than other toolkits, in part because a different solver and model file needs to be separately defined for each set of hyperparameters. Figure 1 provides a snippet of code for the LeNet CNN architecture. The model consists of a 7-layer convolutional network consisting of convolution max pooling and activation layer.

### Deeplearning4j

Deeplearning4j is a multi-platform toolkit with GPU support developed by Andrej Karpathy and written in Java with a Scala API. This is also a mature toolkit (written in Lua) with many examples available on the Internet. This is not heavily used in medical imaging, and use appears to be declining in the medical field. It has good performance and supports multiple GPUs.

### Tensorflow

Tensorflow is a rather new library (at least to public view) that was developed by Google, but already has strong adoption. Performance is good, and supports multiple GPUs and CPUs. Some view it as more difficult to use directly, but tools are addressing this challenge. Tensorflow provides tools for tuning a network and monitoring performance like Tensorboard. It also has an educational tool available as a web app. (http://playground.tensorflow.org/).

### Theano

Theano is a tool for creating networks using symbolic logic, and is written in Python, but takes advantage of the efficient code base of numpy, which improves performance over standard Python. The symbolic approach may be a challenge for some to learn, but Theano is good for building networks, but more challenging to create complete solutions. Theano includes computation of the gradients used in learning as a “free” byproduct of net creation, which may be useful for those wishing to focus more on network architecture than gradient computations. Documentation quality is fair.

### Keras

**Fig. 2:**
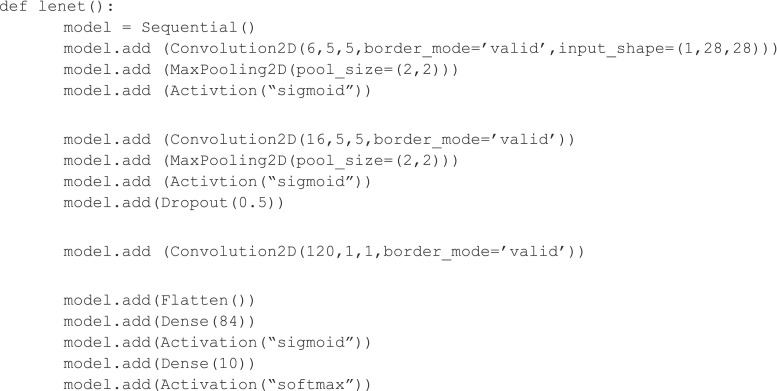
Example code implementing LeNet CNN written in Keras

Keras is a library written in Python that utilizes as backend either Theano or Tensorflow (Fig. 2). It is easier to build complete solutions, and is easy to read, in that each line of code creates one layer of a network. This toolkit seems to have the greatest selection of state-of-the-art algorithms (optimizers, normalization routines, activation functions). Although Keras supports both Theano and Tensorflow backends the assumption for the dimension of the input data is different so careful design is needed in order for the code to be able to work using both back ends. The project is well documented and a set of examples aiming at a wide variety of problems is provided. Pretrained models of commonly used architectures for transfer learning implementation are also provided. At the time of this writing, it was announced that Tensorflow would be adopting Keras as a preferred high-level package. This is not surprising, given that the Keras author—Francois Chollet—is a Google software engineer. Version 2.0 was just announced and promises deeper integration with Tensorflow.

### MXNet

MXNet is a deep learning framework written in C++ with many language bindings, and supports distributed computing, including multi-GPU. It provides access to both lower-level constructs as well as higher/symbolic level API. Performance is considered to be on par with other good systems, including Tensorflow, Caffe, etc. A number of tutorials and training examples are available on GitHub and it has a “model zoo”, which is a collection networks that have been trained on various problems.

### Lasagne

Lasagne is written in Python and is built on top of Theano. It is essentially a thin wrapper to make building networks easier than using Theano directly. As such, its performance largely reflects the underlying performance of Theano.

### Cognitive Network Toolkit (CNTK)

CNTK is developed by Microsoft, and is described as “Visual Studio” for Machine learning. For those that have used Visual Studio for programming, this may be a gentler and more efficient way to get into deep learning. Performance is generally good. It is a rather recent addition to the publicly available toolkits, and usage is currently less than many others.

### DIGITS

DIGITS was developed by NVIDIA, and is a web-based tool for developing deep networks. In many ways, it is like Caffe, and uses a text file not a programming language, to describe the network and parameters. It has a network visualization tool so errors in the text file are more easily identified. In addition, it has tools for visualizing the learning process and has multiple GPU support.

### Torch

Torch is a mature toolkit for machine learning that is written in C. It has good documentation and can be tailored to address specific needs. Because it is written in C, performance is very good.

### PyTorch

**Fig. 3:**
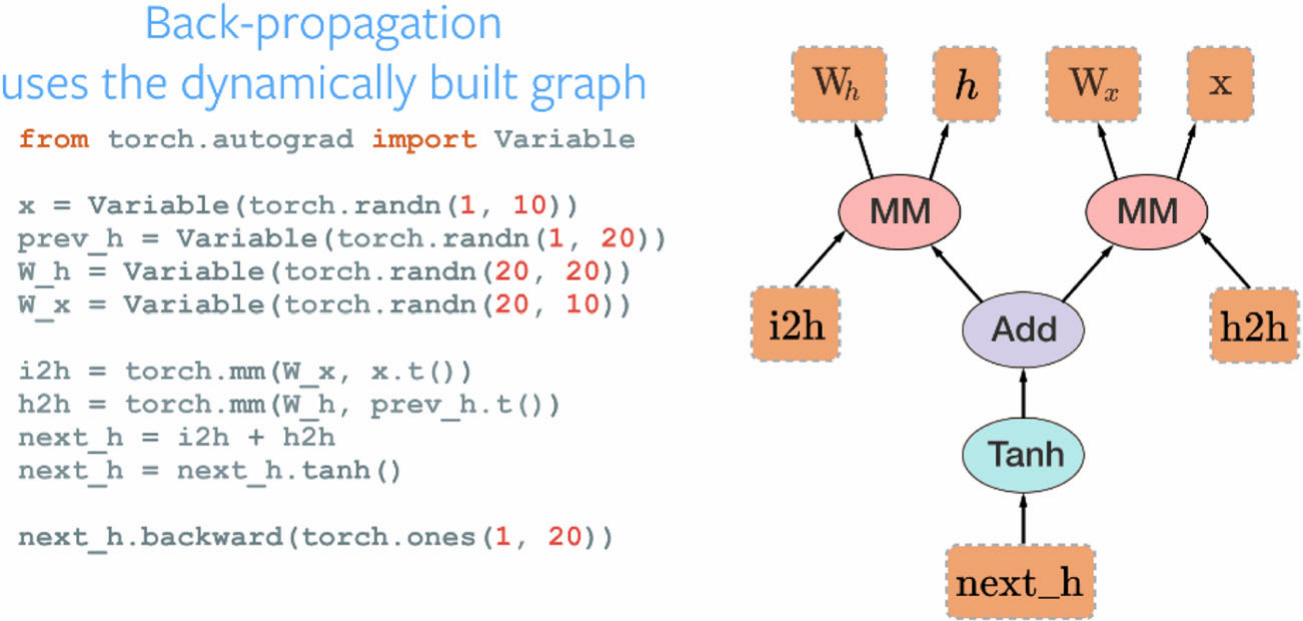
Example of PyTorch code and block diagram equivalent

PyTorch is very recent entry—it was released during the writing of this manuscript. It is a python front end to the Torch computational engine. This should provide the high performance of Torch with good GPU support with a friendlier python front end. The distinction the authors claim is that this more than a wrapper—that there is deep integration to keep points that can allow more flexibility in how the networks are constructed (Fig. 3).

### Pylearn2

Pylearn2 is a machine learning research library developed by Laboratoire d’Informatique des Systèmes Adaptatifs (LISA) at University of Montreal [9]**.** Pylearn2 offers a collection of classical machine learning algorithms as well as deep neural network algorithms written in Python. However, Pylearn2 is not as complete other toolkits such as Keras or MXNet.

### Chainer

Chainer is a bit different from other toolkits because it builds the network as part of its computation. Its authors describe it that most tools are “Define-then-run” which means you define the architecture and then run it. Chainer attempts to build and optimize its architecture as part of the learning process, or as they call it “Define-by-Run.” Chainer stores its computations rather than the programming logic. This allows it to fully leverage the power of Python.

### Other Libraries

Besides the abovementioned libraries that a more broadly utilized, there are more open source solutions that focus on more specific tasks. For instance, Nolearn offers a good implementation of deep belief networks. Sklearn-theano offers a programming syntax that matches the one of scikit-learn (that is the main library for machine learning in Python) to work with the Theano library. Paddle is offering better capabilities for natural language processing, while H2O solutions are oriented to big data analytics offering solutions that combine well with software solutions like Spark.

Table 1 Open source projects ranked based on the stars and forks received by the users.

**Table 1:** Captures the ranking of the open software libraries based on the stars and forks received by the community on GitHub, an online repository for open source projects

Framework	Stars	Forks	Contributors	Language
Caffe	15,057	9338	222	C++
Keras	10,875	10,875	327	Python
MXNet	7471	2764	250	C++
Torch	6163	1793	113	Lua
Convnetjs	6128	1198	15	JavaScript
Deeplearning4j	5090	1970	103	Java
Tensorflow	4505	667	573	Python
Paddle	4069	1024	53	C++
DSSTNE	3531	559	22	C++
Chainer	1983	512	96	Python
DIGITS	1800	1052	34	Python
H2O	1628	714	70	Java

### Comparison of Toolkits

There are a few open efforts to provide benchmarks that compare the performance of these tools. One example can be found at https://github.com/soumith/convnet-benchmarks. This site compares several toolkits with several different CNN-style deep learning networks, including: AlexNet, GoogleNet, and OxfordNet on a specific set of test hardware. Based on their results, Torch is faster than both Tensorflow and Caffe.

Although the majority of the deep neural network libraries are well supported by the online community, not all the libraries support multiple GPUs. The available solutions support parallelization of the computation in multiple GPUs; however, the limiting factor is that the GPUs have to be in the same workstation. Limited support also exists for solutions that can parallelize the computations among different servers (for instance MXNet).

The majority of the libraries support GPU or CPU execution of the code with the CPU solutions being significantly slower. NVIDIA offers a series of GPU cards that support the necessary libraries for deep learning. For rather small neural networks, GPU cards offering 6 GB of RAM are adequate. However, as the models get larger for instance UNET [5] or RESNET [10] the memory requirements significantly increase and GPU cards with 12 or 24Gb RAM should be considered. Currently, the solutions available cover a range of users, from novice to experienced. Tools like NVIDIA DIGITS and deeplearning4j offer good solutions for beginners interested in exploring deep neural networks, suitable for training and educational purposes. On the other hand, libraries like Theano, Tensorflow, and Torch are more appropriate for experienced users who need to have much more control over network architectures. Fortunately, there are libraries that cover the most widely used programming languages.

The open communities behind these libraries offer a wide variety of examples making application of deep neural network models easier. Additionally, Docker- (http://docker.com) based solutions with all the necessary tools are provided for almost all the libraries.

## Conclusions

Writing a deep learning algorithm “from scratch” is probably beyond the skillset of most medical imaging researchers. It is much more efficient to utilize the tremendous resources available in a deep learning toolkit. There are many deep learning toolkits available, and we have described many in this paper. Selecting the best toolkit will depend on the skills and background of the researcher, and may also be impacted by the project and available resources. As such, it is worth spending some time to evaluate available toolkits when a project is begun, to be sure that the best one is chosen for the situation.
